# Latero-cervical lymph node tuberculosis and brachial herpes zoster in a patient diagnosed with HIV infection

**DOI:** 10.1186/1471-2334-14-S4-P39

**Published:** 2014-05-29

**Authors:** Iosif Marincu, Emilian Popovici, Patricia Poptelecan, Sorina Laitin, Nicoleta Bertici, Livius Ţîrnea

**Affiliations:** 1 Department of Infectious Diseases, Pneumology and Parasitology, Dr. Victor Babeş University of Medicine and Pharmacy, Timişoara, Romania

## 

Tuberculosis and Herpes Zoster are known as common opportunistic infections in patients diagnosed with HIV infection.

This is the presentation of a clinical case with right latero-cervical lymph node tuberculosis associated with brachial Herpes Zoster and oral candidiasis in a patient recently diagnosed with HIV infection, stage C3.

The authors present the case of a 23 years old student, admitted in the Clinic of Infectious Diseases Timişoara for a latero-cervical lymphadenopathy in the right side, that appeared simultaneously with a progressively asthenic syndrome associated with weight loss in the past three months, night sweats and loss of appetite. We mention that she also presented a maculo-papular and vesicular rash in the upper limb, accompanied by burning pain. In order to establish the diagnosis, multiple biological samples were collected (blood cell identification, erythrocyte sedimentation rate (ESR), C-reactive protein (CRP), lingual swabs, throat swabs, blood culture, ELISA test for HIV Ab, Western blot test, CD4 lymphocyte, viral load, etc.) and paraclinical investigations (lymph node biopsy, histological examination and cultures for Koch bacilli; cultures for Koch bacilli in the secretions from the bronchial lavage; chest radiography).

From the biological samples we highlight: WBC=3,240/µL, ESR=125 mm/1h, CRP=32.83 mg/L, fibrinogen=3.77 mg%, HIV Ag-Ab=positive, HIV test positive, CD4=13 cells/µL, viral load=320,169 copies/mL; there were no radiological changes in the lungs. The histopathological exam of the lymph node biopsy pleaded for lymph node tuberculosis. The cultures for Koch bacilli in the secretions from the bronchial lavage were positive. The culture for Koch bacilli in the lymph node biopsy was also positive. The lingual swab, using Sabouraud medium culture revealed *Candida albicans*. Putting together the clinical data and the biological samples and the test results, we have established the diagnosis of lymph node tuberculosis, brachial herpes zoster, oral candidiasis, HIV infection stage C3. Under treatment with antituberculous and antiretroviral drugs the evolution of the patient was favorable.

The early diagnosis of the opportunistic infections allows the detection of the patients with HIV infection and the establishment of a specific and efficient therapy.

